# The Role of Executive Function in the Co-occurrence of ADHD and Developmental Dyscalculia in Chinese Children

**DOI:** 10.31083/AP42712

**Published:** 2025-05-28

**Authors:** Jing Zhang, Min Dong, Lu Liu, Sunwei Qiu, Meirong Pan, Xinlin Zhou, Qiujin Qian

**Affiliations:** ^1^Peking University Sixth Hospital/Institute of Mental Health, 100191 Beijing, China; ^2^NHC Key Laboratory of Mental Health (Peking University), National Clinical Research Center for Mental Disorders (Peking University Sixth Hospital), 100191 Beijing, China; ^3^State Key Laboratory of Cognitive Neuroscience and Learning, Faculty of Psychology, Beijing Normal University, 100875 Beijing, China

**Keywords:** ADHD, developmental dyscalculia, executive function, children

## Abstract

**Objective:**

This study aimed to elucidate the characteristics of executive function deficits in children with Attention-deficit/hyperactivity disorder (ADHD) comorbid with developmental dyscalculia (ADHD+DD).

**Methods:**

Three groups of Chinese children (n = 637) aged from 6 to 16 years were included in this study. Initially, a between-group comparison on both performance-based and scale-based executive function was conducted, controlling for age, Raven score, and gender. Partial correlation analysis and regression analysis were then used to investigate the association between executive function, ADHD symptoms, and arithmetic ability. Furthermore, logistic regression analysis and path analysis were used to differentiate the effect of executive functions on ADHD without developmental dyscalculia (ADHD-DD) and ADHD+DD.

**Results:**

Both ADHD groups had more severe executive function impairment than the control group. Compared with the ADHD-DD group, the ADHD+DD group performed worse in performance-based executive functions but similar in scale-based executive functions. ADHD-DD and ADHD were differentiated by inhibition (odds ratio (OR) = 2.00, 95% CI = 1.42; 2.81) and processing speed (OR = 0.90, 95% CI = 0.84; 0.97). In terms of symptom dimensions, verbal working memory had an effect on ADHD symptoms and complex subtraction (*p_Ina_* = 0.006, *p_HI_* = 0.018, *p_CS_* = 0.002), processing speed (*p_Ina_* = 0.002, *p_CS_* = 0.001) and working memory factors influenced inattention and complex subtraction (*p_Ina_* < 0.001, *p_CS_* = 0.001), and inhibition (*p* = 0.004) and cognitive flexibility (*p* = 0.013) contributed uniquely to complex subtraction.

**Conclusion:**

Individuals with ADHD+DD exhibit multiple executive function deficits, with inhibition and processing speed being potential etiological factors. Verbal working memory, processing speed, and working memory factors are shared executive function deficits for ADHD symptoms and arithmetic ability. Cognitive flexibility and inhibition are specific risk factors for arithmetic ability.

## Main Points

- Compared with the ADHD without developmental dyscalculia (ADHD-DD) group, individuals with ADHD with developmental dyscalculia (ADHD+DD) showed worse performance-based executive function but similar scale-based executive function.
- Verbal working memory, processing speed, and scale-based working memory factors are potential shared risk factors for ADHD and arithmetic ability.
- Cognitive flexibility and inhibition have specific contributions to arithmetic ability.
- Processing speed and inhibition are potential ethological elements for distinguishing ADHD-DD and ADHD+DD.

## 1. Introduction

Attention-deficit/hyperactivity disorder (ADHD) and developmental dyscalculia (DD) in childhood affect approximately 5% to 8% of the total population, with comorbidity occurring at a rate of approximately 25% [1, 2]. The comorbidity of ADHD and DD not only exacerbates the academic performance and interpersonal challenges of affected children but also increases the family’s financial burden for medical treatment, educational training, and specialized interventions [3, 4]. However, the neuropsychological characteristics of this comorbidity remain poorly understood. Gaining insight into executive function impairments is essential for developing targeted early treatment goals and improving potential long-term social and developmental outcomes.

Executive function deficits have long been recognized as a significant feature of ADHD [5, 6]. A meta-analysis revealed that individuals with ADHD exhibit multiple deficits in executive function, including impairments in working memory, response inhibition, and cognitive flexibility [7]. These executive functions involve cognitive processes that regulate and guide goal-oriented behaviors through high-order thinking [8]. Barkley suggests that deficits in executive function especially inhibition impairment, are the core impairment of ADHD [5]. Studies have also highlighted other deficits, such as working memory and processing speed, with perceptual and processing aspects more deeply associated with the inattentive subtype [9, 10]. Beyond performance-based executive functions deficit, children with ADHD also exhibit aberrations in scale-based executive function, which affect the management and regulation of daily life behavior [11, 12]. The ADHD combined subtype is likely to be associated with an elevated Behavior Regulation Index (BRI) of the Behavior Rating Inventory of Executive Function (BRIEF) and the inattention type is associated with a higher Metacognition Index (MI) of the BRIEF. This helps supplement the neuropsychological assessment of ADHD [12, 13].

Neuropsychological research on dyscalculia has evolved over time. Traditionally, experts have suggested that learning disorders such as dyscalculia are not related to executive function impairments but rather involve magnitude representation [14, 15]. However, as research has progressed, individuals with dyscalculia have been shown to exhibit distinct executive function deficits, as demonstrated in neuroimaging studies [15, 16]. A study has found that children with dyscalculia exhibit impairments in both verbal working memory and spatial working memory compared with typically developing children [17]. A systemic review reported that children with dyscalculia exhibit slower processing speed for both visual and verbal stimuli than typical children. Additionally, these children also demonstrate poorer performance in inhibition and cognitive flexibility [18]. Another study showed that executive function could effectively differentiate students with dyscalculia from their typically developing peers across different grades. Specifically, processing speed plays a key role in Grade 2, cognitive flexibility in Grade 6, and response inhibition in Grade 10 [19]. These findings suggest that children with dyscalculia not only exhibit executive function deficits but are also affected differently across various developmental stages.

Several neuropsychological studies have compared the executive function between ADHD and ADHD comorbid with learning disability (ADHD+LD), observing that both performance-based executive function and scale-based executive function in ADHD+LD are worse than in controls but not necessarily worse than in ADHD [20, 21, 22].

However, compared with dyslexia, research specifically on the neuropsychological characteristics of ADHD and dyscalculia, as well as on the role of executive function in the relationship between ADHD symptoms and arithmetic skill, has been relatively limited. A limited study on executive function was conducted by rating scale assessments in developmental dyscalculia. The evaluation of scale-based executive function in comorbidity can provide a multidimensional perspective on the executive function characteristics of the comorbidity.

The reasons for the aforementioned limitations are multifaceted. Firstly, the dimensions for assessing dyscalculia are diverse [16, 23]. In China, there are limited standardized assessment methods. Previous diagnostic criteria or assessments were primarily based on students’ academic performance in school, which fails to provide a quantitative assessment of arithmetic skill. The Online Psychological Experiment System (OPES) (http://www.dweipsy.com/lattice/) provides diagnostic tests for learning disabilities and accurate learning ability assessments which are suitable for Chinese primary and secondary school students [24, 25]. This approach helps address the challenges of quantifying assessments of arithmetic skill. Secondly, while number sense abilities are present from birth, children typically receive earlier training in language and reading skills. As a result, when children experience reading difficulties, they tend to be identified easily [26]. Compared with dyscalculia, reading disabilities have a higher comorbidity rate with ADHD, leading to more research being carried out on the executive function of ADHD and dyslexia [2].

Neuropsychological studies have primarily proposed some related hypotheses based on studies of ADHD and dyslexia. A common etiological hypothesis suggests that neurodevelopmental disorders are determined by multiple factors, with some shared risk factors contributing to the occurrence of comorbidity. It was observed that ADHD, reading disorder (RD), and their comorbidities (ADHD+RD) have common executive function deficits, such as processing speed and verbal working memory [27, 28, 29]. The cognitive subtype hypothesis describes comorbidity as characterized not only by an increased susceptibility to both disorders but also by the possibility of more severe impairments [14]. Studies on ADHD and dyslexia have shown that only individuals with comorbidities exhibit severe deficits in rapid naming and have greater deficits in working memory [30, 31]. Both hypotheses have also been supported by neuroimaging research, where they were found to coexist [32, 33].

With respect to ADHD and dyscalculia, Rubinsten and Henik [34] hypothesized cognitive models of comorbid ADHD and DD. The hypotheses describe various possibilities regarding the executive function characteristics of comorbidity, including executive function deficit overlap, exacerbation, or coexistence. One study which focused on ADHD, pure dyscalculia, and their comorbidity, reported that both independent disorders and their comorbidities had a similar degree of verbal working memory deficits, indicating a common etiological hypothesis [35].

In summary, it is essential to explore the executive function profiles of ADHD comorbid with dyscalculia to deepen the understanding of how these cognitive processes manifest and influence such comorbid conditions, particularly in Chinese students. Investigation of the executive function characteristic of the comorbidity will help scientists and educational experts to comprehensively understand affected children and develop efficient and targeted interventions for these children.

Thus, three related hypotheses are proposed in this study:

(1) Individuals with ADHD have worse performance-based executive function and scale-based executive function than controls, while those of ADHD+DD are even worse than those of ADHD-DD.

(2) ADHD symptoms and arithmetic ability are associated with executive functions, with impaired verbal working memory or processing speed being shared risk factors for ADHD symptoms and arithmetic ability.

(3) If the between-group comparisons of the executive functions of ADHD+DD and ADHD-DD show significant differences, some elements of the executive functions could be the etiological factors for the comorbidity and could distinguish ADHD+DD from ADHD-DD.

## 2. Method

### 2.1 Participants

#### 2.1.1 Sample Size

This study is a cross-sectional study with three groups. Analysis of covariance (ANCOVA) was conducted. The sample size formula below was used for the sample size calculation:

$$n=\varphi^{2}\,\left(\sum S_{i}^{2}/g\right)/\left[\sum {\left({\bar{X}}_{ı}-\bar{X}\right)}^{2}/\left(g-1\right)\right]$$

The variable n represents the sample size per group, g refers to the number of groups, X indicates the mean of each group, and S represents the standard deviation of each group.

Statistical significance was set at α = 0.05 (two-tailed) and the power (1-β) was set at 0.90. The minimum required sample size for comparing quantitative data across three independent groups was calculated to be 249 participants in total, meaning that each group must include at least 83 participants.

This study enrolled a total of 507 children with ADHD (ADHD-DD = 357, ADHD+DD = 150). ADHD diagnosis was determined by experienced pediatric psychiatrists based on the criteria of the Diagnostic and Statistical Manual of Mental Disorders-Fourth Edition (DSM-IV). Children with ADHD who met the criteria for developmental dyscalculia (http://www.dweipsy.com/lattice/) were diagnosed with ADHD with developmental dyscalculia (ADHD+DD) [24, 25]. A total of 130 age-matched normal controls were recruited from primary and middle schools.

This study was conducted according to the Declaration of Helsinki and was approved by the Ethics and Clinical Research Committees of Peking University Sixth Hospital ((2016) Ethics review number (15), July 13). Written informed consent was obtained from the guardians of all children before the study began.

#### 2.1.2 Inclusion Criteria

(1) ADHD without developmental dyscalculia (ADHD-DD): ① Met the criteria for ADHD; ② Did not meet the criteria for either dyscalculia or dyslexia; ③ Aged from 6–16 years old; and ④ Had a Wechsler Intelligence Scale-intelligence quotient (WISC-IQ) score of >70.

(2) ADHD with developmental dyscalculia (ADHD+DD): ① Met the criteria for ADHD; ② Met the criteria for dyscalculia according to a simple subtraction test from the Online Psychological Experiment System (OPES) (http://www.dweipsy.com/lattice/) [24, 25]; ③ Met or did not meet the criteria of dyslexia according to a word semantic test from the same website; ④ Aged from 6–16 years old; and ⑤ Had a WISC-IQ score of >70.

(3) Normal control (NC): ① Did not meet the criteria for ADHD; ② Did not meet the criteria for either dyscalculia or dyslexia; ③ Aged from 6–16 years old; and ④ Had a WISC-IQ score of >70.

#### 2.1.3 Exclusion Criteria

Children who fulfilled at least one of the following conditions were excluded: ① Had a serious mental disorder such as childhood schizophrenia, autism spectrum disorder, or intellectual disability; ② Had a significant physical or neurological disease or severe sensory impairment that significantly affected task performance; ③ Had a history of any central nervous system stimulant use, including atomoxetine, antipsychotic medications, or antidepressant medications; and ④ Lacked the ability to operate a computer.

### 2.2 Measurement

All the participants and their guardians were required to complete relevant learning tasks, executive function assessments, the WISC-IQ test, and Raven’s Standard Progressive Matrices (R’SPM). Their guardians were asked to finish the diagnosis interview and the rating score evaluation for the participants. For comprehensive measurements, see **Supplementary Material 1**.

The Wechsler Intelligence tasks included assessments of executive functions. Using the WISC-IQ score as a control variable might have led to overcorrection and distortion of the results; therefore, we used the raw score of the R’SPM as the control variable [36].

### 2.3 Procedure

All eligible subjects completed the study-related measurement as mentioned above in a quiet environment. All examiners received relevant training. For the time-limited learning-related tasks, corrected scores were calculated by subtracting the number of incorrect responses from the number of correct responses for the time-limited tasks; this was done to control for the effect of guessing. The standard Z scores for each participant were calculated based on the corrected scores [37].

### 2.4 Statistical Analysis

Data processing and statistical analyses were performed using IBM SPSS Statistics for Windows (Version 23.0. IBM Corp., Armonk, NY, USA) and IBM SPSS Amos for Windows (Version 24.0. IBM Corp., Armonk, NY, USA). Figures were generated using GraphPad Prism for Windows (Version 8.0.0, GraphPad Software, San Diego, CA, USA). A normality test was conducted on the data. Data with a mild positive skew, such as those derived from the ‘colorful Chinese characters task of the Stroop Color-Word Test’, were transformed using square root transformation. For data with a moderate skew, including those derived from the Trail making tasks, task 1 to task 3 of the Stroop Color-Word Test, factors of inhibition (IB), shifting (SFT), and emotional control (ECTRL), logarithmic transformation (lg10 or ln) was used. For data with a moderate negative skew, or data with a both positive and negative skew in different groups, such as those derived from factors of planning/organization (PO), organization of material (OM) and monitor (MONI), and forgotten structure score, rank transformation was applied. For data containing zeros, including those derived from the verbal-span backward, Spatial Span Test (WMS-III)-forward, the detail of immediate scores of the Rey-Osterrieth Complex Figure (Reydi), and the detail of delayed score (Reydd) and derived variables, the method of adding a constant to the original data before taking the square root transformation was applied. After normality transformation, some data still showed mild skewness (skewness values between 2 and 4), which may be attributed to the differences between healthy controls and patients.

For the demographic data, Chi-squared analysis was used to compare category variables and one-way analysis of variance (ANOVA) was conducted for variables, except for R’SPM raw scores, where a non-parametric test (Kruskal-Wallis test) was adopted. Using age (months), gender, and R’SPM raw scores (Raven) as covariates, ANCOVA was conducted to compare all-type executive functions among groups. Post hoc pairwise comparisons were performed using Bonferroni correction. For the performance-based tasks, including the WMS-II, Rey-Osterrieth Complex Figure (ROCF), Trail Making Test (TMT), and Stroop, factor analysis was conducted, resulting in four factors representing spatial working memory (SWM), visual working memory (VisualWM), cognitive flexibility (CF), and inhibition (Inh). ANCOVA and post hoc analysis were also performed for these four factors. Subsequently, partial correlation analysis and multiple regression analysis were performed for all participants, recording the R^2^, F, ΔF, ΔR^2^, and corrected R^2^ values of the multiple regression analysis. Finally, logistic regression and path analysis were employed for the two ADHD groups to examine the differences in executive function between the two groups and explore the underlying mechanisms of executive function of ADHD+DD and ADHD-DD.

## 3. Results

Demographic data are shown in Table 1. Notable differences were observed in gender (*p* < 0.001) and age *(p* < 0.001) among the three groups. There were significant differences in both IQ test scores (ADHD+DD < ADHD-DD < NC, all *p* < 0.001) and R’SPM scores (ADHD+DD < ADHD-DD < NC, all *p* < 0.001). Approximately 30.18% of ADHD children suffered from other mental disorders. The differences in other comorbidities were insignificant between the ADHD-DD and ADHD+DD groups (*p* = 0.489) (**Supplementary Material 2**).

**Table 1.** **Demographic characteristics of three groups**.

Object		ADHD+DD	ADHD-DD	NC	*p*-value	*post hoc*
		(n = 150)	(n = 357)	(n = 130)		
Age (months)		110.43 ± 20.56	120.04 ± 28.58	120.53 ± 20.80	<0.001	3, 2 > 1
Gender (boys) (%)		125 (83.30%)	288 (80.70%)	59 (45.40%)	<0.001	/
WISC-IQ		101.57 ± 14.24^a^	109.93 ± 14.14	116.69 ± 10.07	<0.001	3 > 2 > 1
Raw R’SPM scores		36 (27, 43)^b^	43 (36.5, 48)	48 (43, 53)	<0.001^c^	3 > 2 > 1
ADHD subtype					<0.001	/
	ADHD-I	92	222	0	0.857	/
	ADHD-C	58	135	0		/
Comorbidities		42	111	0	0.489	/
Simple subtraction		11.49 ± 8.34	32.72 ± 9.63	38.03 ± 9.03	<0.001	3 > 2 > 1
Complex subtraction		4.73 ± 7.02	11.55 ± 7.82	16.84 ± 7.66	<0.001	3 > 2 > 1
Word semantic test		12.61 ± 11.11	21.91 ± 9.17	28.25 ± 8.45	<0.001	3 > 2 > 1
Graded reading achievement		9.30 ± 8.23	16.93 ± 10.61	23.56 ± 8.70	<0.001	3 > 2 > 1
ADHD RS-IV						
	Inattention	17.89 ± 3.69	17.67 ± 3.75	6.96 ± 3.25	<0.001	3 < 2, 1
	HI	12.36 ± 5.93	11.60 ± 6.25	5.10 ± 3.10	<0.001	3 < 2, 1
	Total	30.25 ± 8.01	29.28 ± 8.15	12.06 ± 5.65	<0.001	3 < 2, 1

Note: ^a^Mean ± SD, ^b^Median (Q1, Q3), ^c^Non-parametric tests analysis. Abbreviations: ADHD, attention-deficit/hyperactivity disorder; ADHD-I, ADHD predominantly inattention type; ADHD-C, ADHD combined type; DD, developmental dyscalculia; NC, normal control; WISC-IQ, Wechsler Intelligence Scale-intelligence quotient; R’SPM, Raven’s Standard Progressive Matrices; ADHD RS-IV, ADHD Rating Scale-IV; HI, hyperactivity/impulsivity; ADHD+DD, ADHD with developmental dyscalculia; ADHD-DD, ADHD without developmental dyscalculia.

Regarding ADHD symptoms, both ADHD groups had significantly higher scale scores compared with the control group (all *p* < 0.001). No significant differences were observed between the two ADHD groups (all *p* > 0.05). The results of the simple subtraction and complex subtraction tests were significantly different among three groups (*p* < 0.001), with the ADHD+DD group showing the worst performance (ADHD+DD < ADHD-DD < NC, all *p* < 0.001). Similar results were obtained for the word semantic test and graded reading achievement test (ADHD+DD < ADHD-DD < NC, all *p* < 0.001).

### 3.1 Between-group Comparison

**Fig. 1.**
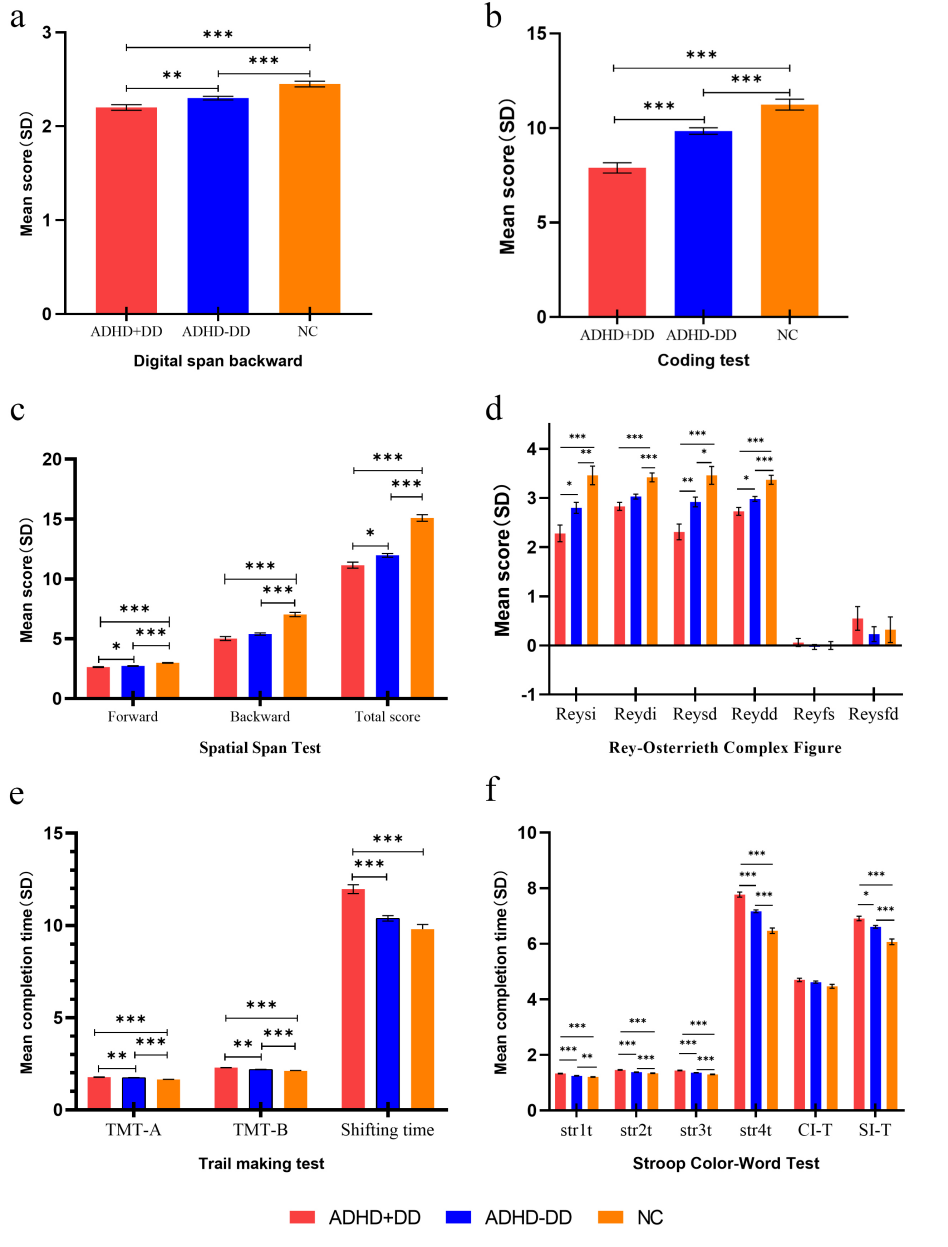
**ANCOVA of performance-based executive function tasks**. (a) Digital span backward. (b) Coding test. (c) Spatial span test. (d) Rey-Osterrieth Complex Figure. (e) Trail making test. (f) Stroop Color-Word Test. Note: ^*⁣**^p < 0.001, ^**^p < 0.01, ^*^p < 0.05. The results are presented as estimated marginal means ± standard errors, with gender, age, and Raven’s Standard Progressive Matrices included as covariates. TMT, Trail Making Test; Str1t, pure Chinese character reading; Str2t, color reading; Str3t, colored Chinese character reading; Str4t, color of the colorful Chinese characters; CI-T, color interference time; SI-T, semantic interference time; Reydd, detail of delayed scores; Reydi, detail of immediate scores; Reysd, structure of delayed scores; Reysi, structure of immediate scores; Reyfd, forgotten detail score; Reyfs, forgotten structure score; ANCOVA, analysis of covariance.

The outcomes of ANCOVA of performance-based executive function are illustrated in Fig. 1a–f. Forgotten structural, forgotten detail, and color interference time were not significantly different among the three groups (*p* > 0.05). ADHD+DD children performed worse than controls in all residual tasks (*post hoc comparison*: all *p* < 0.001). The results of the performance-based tasks, except shifting time (*p* = 0.164), in ADHD-DD children were also inferior to those in controls (*post hoc comparison*: *p*_structure of immediate score(Reysi)_ = 0.007, *p*_structure of delayed score(reysd)_ = 0.025, *p*_T⁢M⁢T-B_ = 0.001, *p*_Pure Chinese characters_ = 0.001, all others *p* < 0.001) but superior to those in ADHD+DD children for the majority of tasks (*post hoc comparison*: *p*_verbal-span backward_ = 0.002, *p*_SWM-forwad_ = 0.018, *p*_SWM-total_ = 0.018, *p*_𝑅𝑒𝑦𝑠𝑖_ = 0.031, *p*_𝑅𝑒𝑦𝑠𝑑_ = 0.005, *p*_detail of delayed score(Reydd)_ = 0.022, *p*_TMT-A_ = 0.006, *p*_semantic Interference Time_ = 0.007, the *p*-value of coding, TMT-B, tasks 1 to 4 of Stroop < 0.001). The rest of the results were not significant (all *p* > 0.05) (**Supplementary Material 3**).

**Fig. 2.**
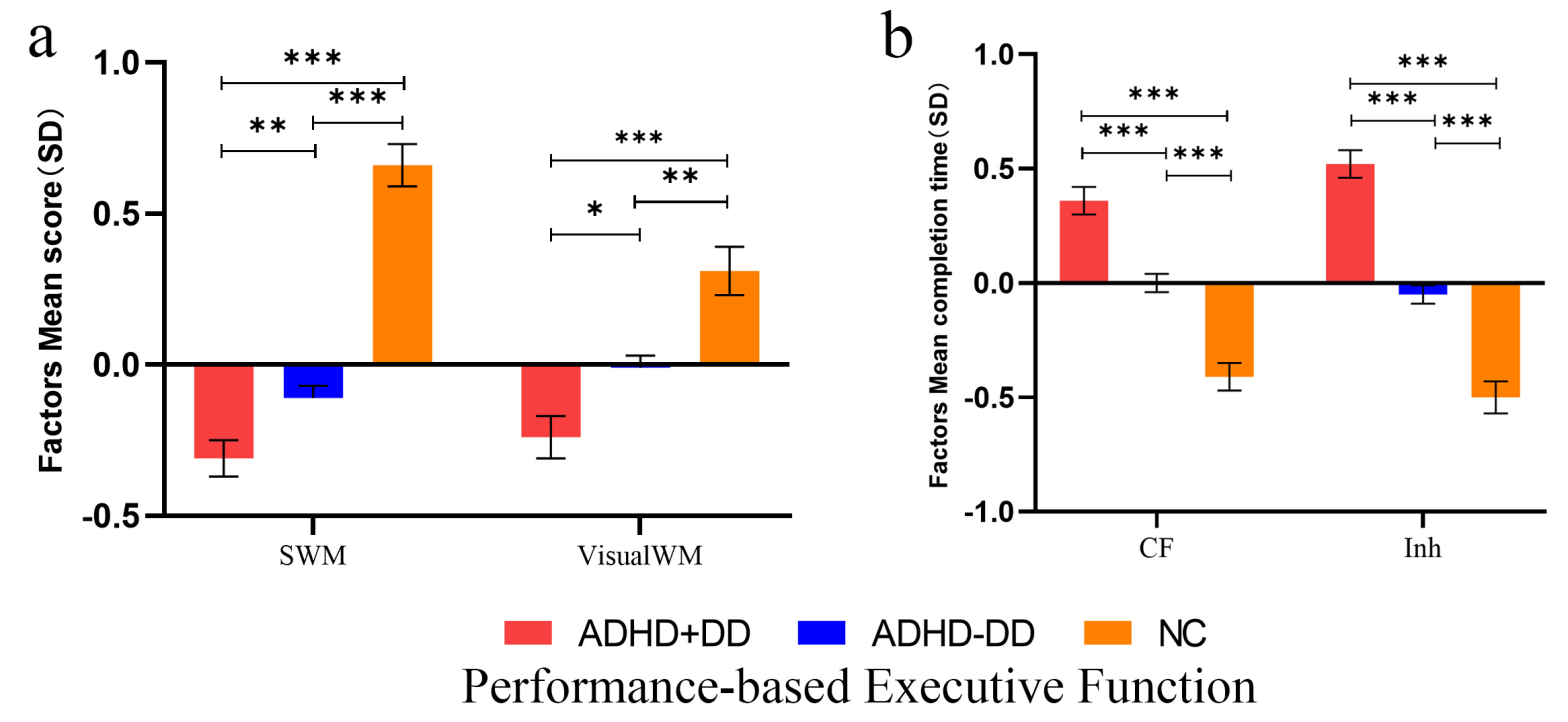
**Between-group comparison of performance-based executive functions**. (a) ANCOVA of the spatial working memory and visual working memory. (b) ANCOVA of cognitive flexibility and inhibition. Note: ^*⁣**^p < 0.001,^**^p < 0.01, ^*^p < 0.05. The results are presented as estimated marginal means ± standard errors, with gender, age, and R’SPM included as covariates. SWM, Spatial working memory; VisualWM, visual working memory; CF, cognitive flexibility; Inh, inhibition.

Four factors were extracted for further analysis, including SWM, VisualWM, CF, and Inh. Factor analysis was not conducted on verbal working memory and processing speed, as each contained only one factor. ANCOVA was conducted to compare the factors of performance-based executive function among the three groups (as shown in Fig. 2a,b and** Supplementary Material 3**). The ADHD+DD group had lower scores on spatial working memory and visual working memory, and longer completion times on cognitive flexibility and inhibition compared with the control group (*post hoc comparison*: all *p* < 0.001) and the ADHD-DD group (*post hoc comparison*: *p*_𝑆𝑊𝑀_ = 0.016, *p*_𝑣𝑖𝑠𝑢𝑎𝑙𝑊𝑀_ = 0.012, *p*_𝐶𝐹_ < 0.001, *p*_𝐼𝑛ℎ_ < 0.001).

**Fig. 3.**
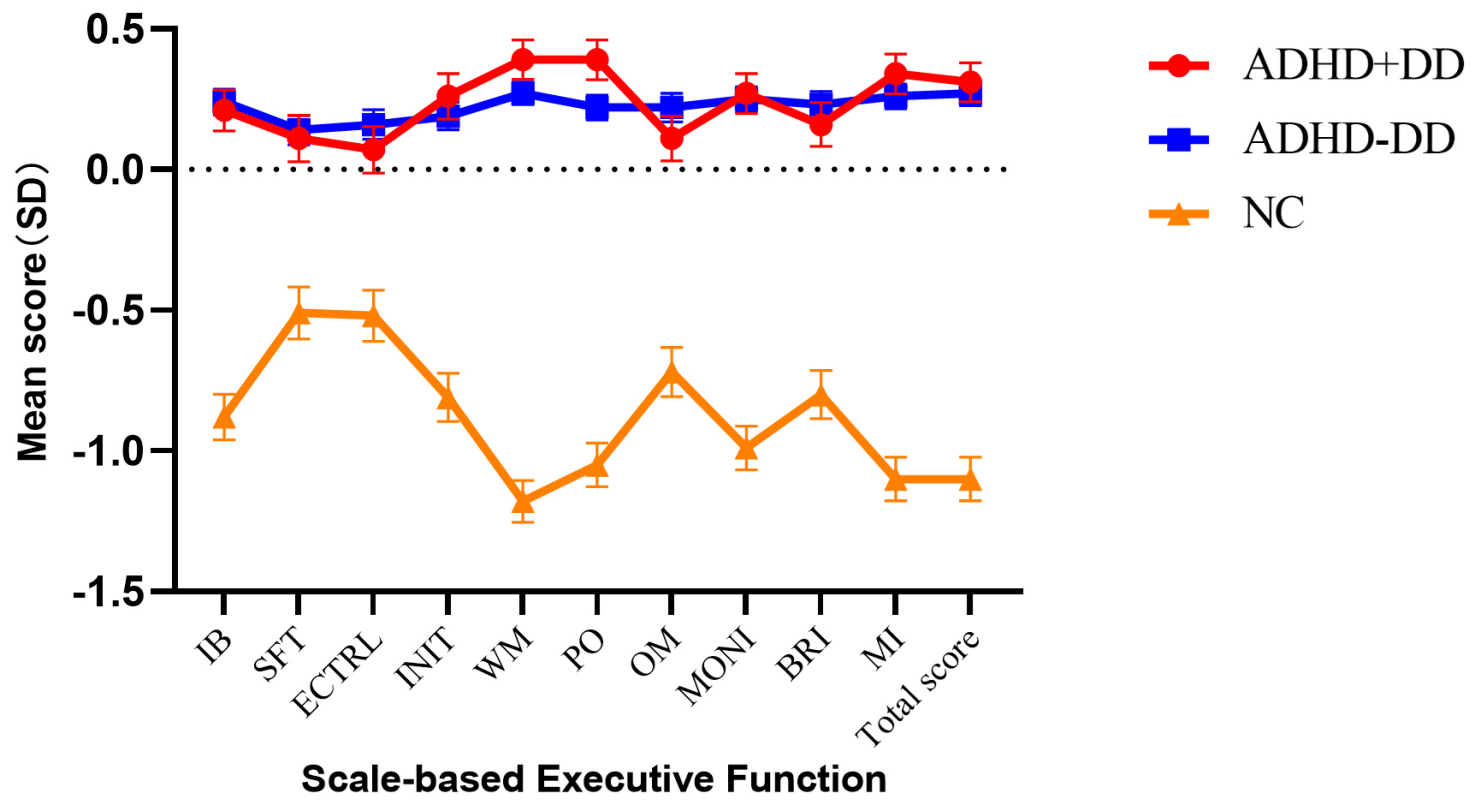
**ANCOVA of scale-based executive functions**. For better readability, z-transformation was applied. The results are presented as estimated marginal means ± standard errors, with gender, age, and Raven’s Standard Progressive Matrices included as covariates. IB, inhibition; SFT, shifting; ECTRL, emotional control; INIT, initiation; WM, working memory; PO, planning/organization; OM, organization of materials; MONI, monitor; BRI, Behavioral Regulation Index; MI, Metacognition Index.

The results of ANCOVA of scale-based executive function are shown in Fig. 3. The MI encompasses the factors of initiation (INIT), working memory (WM), PO, OM, and MONI, while the BRI includes IB, SFT, and ECTRL. The results showed minimal differences in BRIEF scores between the ADHD+DD group and ADHD-DD group (*post hoc comparison*: all *p* > 0.05). Both ADHD groups obtained higher scores than the NC group (*post hoc comparison*: all *p* < 0.001) (**Supplementary Material 3**).

### 3.2 Partial Correlation Analysis

The results of partial correlation analysis among the three groups with gender, age, and Raven scores as covariates for performance-based executive functions or scale-based executive functions are shown separately in Tables 2,3.

**Table 2.** **Partial correlation analysis between performance-based executive functions, ADHD symptoms, and arithmetic ability**.

	CS	Ina	HI	VWM	SWM	CF	Inh	VisualWM	PS
1	1	(*p* < 0.001)	(0.002)	(*p* < 0.001)	(*p* < 0.001)	(*p* < 0.001)	(*p* < 0.001)	(0.002)	(*p* < 0.001)
2	–0.222	1	(*p* < 0.001)	(*p* < 0.001)	(*p* < 0.001)	(*p* < 0.001)	(*p* < 0.001)	(0.001)	(*p* < 0.001)
3	–0.120	0.487	1	(0.007)	(0.001)	(0.790)	(0.576)	(0.009)	(0.592)
4	0.222	–0.189	–0.108	1	(*p* < 0.001)	(*p* < 0.001)	(*p* < 0.001)	0.241	0.005
5	0.202	–0.293	–0.126	0.206	1	(*p* < 0.001)	(*p* < 0.001)	(*p* < 0.001)	(*p* < 0.001)
6	–0.271	0.187	0.011	–0.214	–0.343	1	(*p* < 0.001)	(*p* < 0.001)	(*p* < 0.001)
7	–0.289	0.189	0.022	–0.333	–0.221	0.431	1	(*p* < 0.001)	(*p* < 0.001)
8	0.120	–0.131	–0.104	0.047	0.183	–0.178	–0.139	1	(*p* < 0.001)
9	0.259	–0.217	–0.021	0.112	0.200	–0.351	–0.381	0.153	1

Note: n = 637 (ADHD-DD = 357, ADHD+DD = 150, and NC = 130). Covariables: gender, age, and raw score of Raven’s Standard Progressive Matrices. Significance levels are displayed in the upper triangle. The lower triangle shows the correlation coefficients. Abbreviations: CS, complex subtraction; VWM, verbal working memory; SWM, spatial working memory; CF, cognitive flexibility; Inh, inhibition; PS, processing speed; VisualWM, visual working memory.

**Table 3.** **Partial correlation analysis between scale-based executive function, ADHD symptoms, and arithmetic ability**.

	CS	Ina	HI	IB	SFT	ECTRL	INIT	WM	PO	OM	MONI
1	1	(*p* < 0.001)	(0.002)	(0.095)	(0.145)	(0.148)	(0.004)	(*p* < 0.001)	(*p* < 0.001)	(0.098)	(0.070)
2	–0.222	1	(*p* < 0.001)	(*p* < 0.001)	(*p* < 0.001)	(*p* < 0.001)	(*p* < 0.001)	(*p* < 0.001)	(*p* < 0.001)	(*p* < 0.001)	(*p* < 0.001)
3	–0.120	0.487	1		(*p* < 0.001)	(*p* < 0.001)	(*p* < 0.001)	(*p* < 0.001)	(*p* < 0.001)	(*p* < 0.001)	(*p* < 0.001)
4	–0.066	0.486	0.678	1	(*p* < 0.001)	(*p* < 0.001)	(*p* < 0.001)	(*p* < 0.001)	(*p* < 0.001)	(*p* < 0.001)	(*p* < 0.001)
5	–0.058	0.316	0.175	0.427	1	(*p* < 0.001)	(*p* < 0.001)	(*p* < 0.001)	(*p* < 0.001)	(*p* < 0.001)	(*p* < 0.001)
6	–0.058	0.317	0.323	0.579	0.602	1	(*p* < 0.001)	(*p* < 0.001)	(*p* < 0.001)	(*p* < 0.001)	(*p* < 0.001)
7	–0.114	0.487	0.199	0.445	0.591	0.492	1	(*p* < 0.001)	(*p* < 0.001)	(*p* < 0.001)	(*p* < 0.001)
8	–0.195	0.667	0.334	0.529	0.458	0.401	0.644	1	(*p* < 0.001)	(*p* < 0.001)	(*p* < 0.001)
9	–0.178	0.613	0.299	0.518	0.487	0.412	0.665	0.755	1	(*p* < 0.001)	(*p* < 0.001)
10	–0.066	0.493	0.302	0.444	0.326	0.338	0.482	0.610	0.575	1	(*p* < 0.001)
11	–0.072	0.603	0.483	0.709	0.404	0.416	0.566	0.644	0.662	0.575	1

Note: n = 637 (ADHD-DD = 357, ADHD+DD = 150, and NC = 130). Covariables: gender, age, and raw score of Raven’s Standard Progressive Matrices. Upper triangle represents the significance of the correlations, while the lower represents correlation coefficients.

A significant negative correlation was observed in terms of ADHD core symptoms and arithmetic ability (*r*_Ina-CS_ = –0.222 (*p* < 0.001), *r*_HI-CS_ = –0.120 (*p* = 0.002)).

There were low to moderate correlations among the performance-based executive functions (|*r*| = 0.112~0.431, *p*_VWM-PS_ = 0.005, other *p* < 0.001), except for the correlation between verbal working memory and visual working memory (VisualWM) (*r* = 0.047, *p* = 0.241). All the performance-based executive functions were significantly correlated with arithmetic ability and inattention score (|*r*| = 0.120~0.293, *p*_CS-visualWM_ = 0.002, *p*_ina-visualWM_ = 0.001, other *p* < 0.001). Verbal working memory, spatial working memory, and visual working memory were negatively correlated with HI symptoms (*r*_𝑆𝑊𝑀_ = –0.126, *p* = 0.001; *r*_𝑉𝑊𝑀_ = –0.108, *p* = 0.007; *r*_𝑉𝑖𝑠𝑢𝑎𝑙𝑊𝑀_ = –0.104, *p* = 0.009).

The scale-based executive function factors positively correlated with each other (*r* = 0.326~0.755, all *p* < 0.001). INIT, WM, and PO were negatively correlated with complex subtraction (*r* = –0.114~–0.195, *p*_𝐼𝑁𝐼𝑇_ = 0.004, *p*_𝑊𝑀_ < 0.001), *p*_𝑃𝑂_ < 0.001). All factors were positively correlated with ADHD symptoms (*r* = 0.175~0.678, all *p* < 0.001).

### 3.3 Multiple Regression Analysis

Further analysis was conducted to explore the role of executive function in ADHD symptoms and learning ability dimension. The results of the impacts of executive functions on complex subtraction and ADHD symptoms are shown in Tables 4,5,6.

**Table 4.** **Multiple regression analysis of the effect on arithmetic ability**.

Subject	Step 1		Performance-based	Step 2-1		Scale-based	Step 2-2	
	β	*p*-value	EF	β	*p*-value	EF	β	*p*-value
Constant		<0.001	Constant		0.002	Constant		0.062
Gender	0.11	0.001	Gender	0.05	0.109	Gender	0.07	0.033
Age	0.34	<0.001	Age	0.13	0.002	Age	0.35	<0.001
Raven	0.33	<0.001	Raven	0.13	<0.001	Raven	0.29	<0.001
			VWM	0.12	0.002	IB	0.00	0.970
			SWM	0.08	0.060	SFT	0.04	0.342
			VisualWM	0.04	0.262	ECTRL	–0.01	0.822
			PS	0.12	0.001	INIT	0.01	0.914
			Inh	–0.14	0.004	WM	–0.19	0.001
			CF	–0.13	0.013	PO	–0.13	0.025
						OM	0.07	0.127
						MONI	0.09	0.123
R^2^	0.345			0.443			0.381	
Corrected R^2^	0.342			0.435			0.370	
F	<0.001			<0.001			<0.001	
ΔR^2^	0.345			0.098			0.035	
ΔF	<0.001			<0.001			<0.001	

Note: n = 637 (ADHD-DD = 357, ADHD+DD = 150, and NC = 130). Abbreviations: β, standardized coefficients; SE, standard error; EF, executive function.

**Table 5.** **Multiple regression analysis of the effect on inattention**.

Subject	Step 1		Performance-based	Step 2-1		Scale-based	Step 2-2	
	β	*p*-value	EF	β	*p*-value	EF	β	*p*-value
Constant		<0.001	Constant		<0.001	Constant		0.043
Gender	–0.23	<0.001	Gender	–0.18	<0.001	Gender	–0.04	0.186
Age	0.09	0.031	Age	0.28	<0.001	Age	0.07	0.028
Raven	–0.25	<0.001	Raven	–0.03	0.597	Raven	–0.10	0.002
			VWM	–0.13	0.006	IB	0.06	0.165
			SWM	–0.26	<0.001	SFT	–0.06	0.107
			VisualWM	–0.07	0.135	ECTRL	0.00	0.933
			PS	–0.13	0.002	INIT	0.01	0.783
			Inh	0.05	0.387	WM	0.38	<0.001
			CF	0.02	0.740	PO	0.16	0.001
						OM	0.04	0.294
						MONI	0.21	<0.001
R^2^	0.114			0.231			0.566	
Corrected R^2^	0.110			0.220			0.558	
F	<0.001			<0.001			<0.001	
ΔR^2^	0.114			0.117			0.451	
ΔF	<0.001			<0.001			<0.001	

Note: n = 637 (ADHD-DD = 357, ADHD+DD = 150, and NC = 130).

**Table 6.** **Multiple regression analysis of the effect on hyperactivity/impulsivity**.

Subject	Step 1		Performance-based	Step 2-1		Scale-based	Step 2-2	
	β	*p*-value	EF	β	*p*-value	EF	β	*p*-value
Constant		<0.001	Constant		<0.001	Constant		<0.001
Gender	–0.21	<0.001	Gender	–0.20	<0.001	Gender	–0.04	0.142
Age	–0.16	<0.001	Age	–0.09	0.100	Age	–0.04	0.193
Raven	–0.13	0.002	Raven	–0.04	0.394	Raven	–0.04	0.212
			VWM	–0.12	0.018	IB	0.72	<0.001
			SWM	–0.14	0.008	SFT	–0.09	0.025
			VisualWM	–0.11	0.024	ECTRL	–0.02	0.566
			PS	0.00	0.947	INIT	–0.10	0.015
			Inh	–0.03	0.230	WM	0.05	0.341
			CF	–0.07	0.579	PO	–0.04	0.401
						OM	0.03	0.376
						MONI	0.06	0.231
R^2^	0.120			0.150			0.546	
Corrected R^2^	0.116			0.138			0.538	
F	<0.001			<0.001			<0.001	
ΔR^2^	0.120			0.029			0.426	
ΔF	<0.001			0.002			<0.001	

Note: n = 637 (ADHD-DD = 357, ADHD+DD = 150, and NC = 130).

Firstly, all covariates were used as dependent variables in Step 1, with all three models being significant (*p* < 0.001). Then, in Steps 2-1 or 2-2, performance-based executive functions or scale-based executive functions were added to the model based on Step 1 to assess the overall effect of the model on the dependent variables. The results showed that all models remained significant (*p* < 0.001).

As shown in Table 4, after executive functions were added to the model, R^2^ increased, indicating that performance-based executive function contributed an additional 9.8% (*p* < 0.001) and scale-based executive function provided 3.5% (*p* < 0.001) extra effect on arithmetic ability. This highlights the independent contribution of executive functions to the dependent variables. Similar findings were observed for inattention (11.7% additional effect of performance-based executive function, *p* < 0.001 and 45.1% additional effect of scale-based executive function, *p* < 0.001) and hyperactivity/impulsivity, with 2.9% extra effect explained by performance-based executive functions (*p* = 0.002) and 42.6% by scale-based executive function (see Tables 4,5,6).

The effects of verbal working memory on both ADHD symptoms and complex subtraction were significant (β_𝐼𝑛𝑎_ = –0.13, *p* = 0.006; β_𝐻𝐼_ = –0.12, *p* = 0.018; β_𝐶𝑆_ = 0.12, *p* = 0.002). Processing speed exhibited notable effects on inattention symptoms (β = –0.13, *p* = 0.002) and complex subtraction (β = 0.12, *p* = 0.001). Both ADHD symptoms were affected by spatial working memory (β_𝐼𝑛𝑎_ = –0.26, *p* < 0.001; β_𝐻𝐼_ = –0.14, *p* = 0.008). Inhibition (β = –0.14, *p* = 0.004) and cognitive flexibility (β = –0.13, *p* = 0.013) made specific contributions to complex subtraction. The result of visual working memory on hyperactivity/impulsivity was also significant (β = –0.13, *p* = 0.013).

WM (β_𝐼𝑛𝑎_ = 0.38, *p* < 0.001; β_𝐶𝑆_ = –0.19, *p* = 0.001) and PO (β_𝐼𝑛𝑎_ = 0.16, *p* = 0.001; β_𝐶𝑆_ = –0.13, *p* = 0.025) had an effect on inattention symptoms and complex subtraction. MONI (β = 0.21, *p* < 0.001) was specific to inattention symptoms, while IB (β = 0.72, *p* < 0.001), SFT (β = –0.09, *p* = 0.025), and INIT (β = –0.10, *p* = 0.015) were specific to hyperactivity/impulsivity.

### 3.4 Logistic Regression Analysis and Path Analysis

To further elucidate the impact of executive functions on different ADHD groups, logistic regression analysis was conducted. The two ADHD groups were considered as the dependent variable, with the ADHD-DD group serving as the reference category. Gender (with male as the reference) alone did not distinguish between these two groups. Finally, Raven scores, age, and all performance-based executive function factors were included in the regression analysis as independent variables.

The model demonstrated a favorable goodness of fit (Hosmer-Lemeshow test *p* > 0.05). Inhibition (OR = 2.00, 95% CI = 1.42; 2.81) and processing speed (OR = 0.90, 95% CI = 0.84; 0.97) had significant effects in differentiating ADHD+DD from ADHD-DD. As the processing speed score decreased by 1 unit, the probability of an ADHD+DD diagnosis increased by 9.0%. For each point increase in inhibition reaction time, the likelihood of ADHD+DD was 2.0 times that of ADHD-DD. All other executive functions were meaningless for discriminating ADHD patients with or without DD (all *p* > 0.05) (**Supplementary Material 4**).

**Fig. 4.**
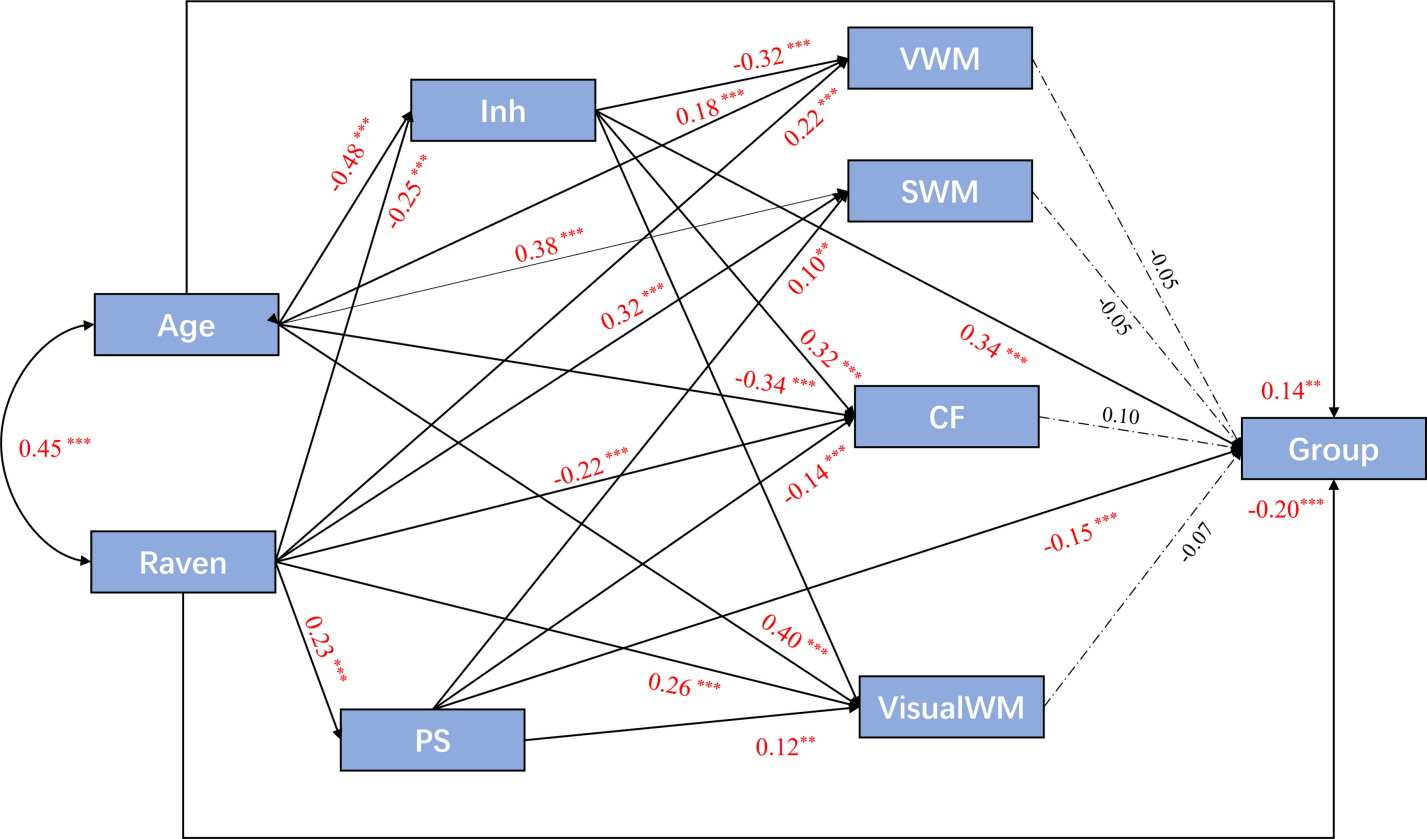
**Path analysis of performance-based executive functions and two categories of ADHD**. Factor loadings are standardized, n = 507. The model fits the data well (GFI = 0.942, NFI = 0.922, CFI = 0.929, RMSEA = 0.129). All insignificant connectors were deleted, except the effect of the intermediate variables on group. ^**^: *p* < 0.01, ^*⁣**^: *p* < 0.001. GFI, Goodness of Fit Index; NFI, Normed Fit Index; CFI, Comparative Fit Index; RMSEA, Root Mean Square Error of Approximation.

Path analysis was conducted to elucidate the influence of individual executive functions and to illustrate their contribution to ADHD+DD. Spatial working memory, cognitive flexibility, visual working memory, and verbal working memory were found to be insignificant in the logistic regression analysis and were therefore designated as intermediate variables. Meanwhile, processing speed and inhibition were identified as independent variables, while the dependent variables remained unchanged. The primary result is illustrated in Fig. 4.

Only processing speed and Inh demonstrated significant total effects and direct effects in distinguishing these two groups (E_total-PS_ = –0.15, *p* = 0.001; E_total-Inh_ = 0.34, *p* = 0.001), while both indirect effect and other effects of performance-based executive function factors were insignificant (all *p* > 0.05).

The results of mediation analysis in the path analysis were consistent with those from the regression analysis, showing that only processing speed and inhibition had significant total and direct effects on group (E_direct-PS_ = –0.15, *p* = 0.001; E_direct-Inh_ = 0.34, *p* = 0.001). Conversely, the indirect effects through intermediate variables were not significant (all *p* > 0.05) (**Supplementary Material 4**).

## 4. Discussion

This study was performed to investigate the characteristics of executive function in Chinese children with ADHD+DD. Overall, it was observed that the individuals with ADHD+DD had multiple deficits in both performance-based executive functions and scale-based executive functions, as did the individuals with ADHD-DD. Both ADHD symptoms and arithmetic ability were commonly affected by verbal working memory, processing speed, and WM factors of scale-based executive functions. Inhibition and processing speed was able to discriminate individuals with ADHD-DD from those with ADHD+DD.

### 4.1 Executive Function Characteristics of Children With ADHD+DD

As showed in previous research, individuals with ADHD exhibit deficits in performance-based executive functions. Neuropsychological studies suggest that executive function is a critical endophenotype of ADHD [6]. Various studies consistently report that individuals with ADHD have deficits in inhibition, working memory, and cognitive flexibility [38, 39]. The present study also found that the scores of each factor of scale-based executive function of both ADHD groups were significantly higher than those of the control group. The BRIEF scale accurately reflects children’s executive function performance in daily life, addressing the limitations of performance-based executive function tests, which may be constrained by their structured environments and tasks [11, 12]. In the present study, the ADHD+DD group exhibited poorer performance in performance-based executive functions but not in scale-based assessments compared with the ADHD-DD group. Several studies support the existence of executive function deficits in individuals with dyscalculia, especially in performance-based executive functions, such as verbal working memory, processing speed, and inhibition [18, 23]. However, specific research on scale-based executive function in dyscalculia is limited. A cross-sectional study conducted in China reported that people with ADHD+LD have more severe executive function in both performance-based and scale-based functions. The differences in scale-based executive functions in the present study might be due to differences in diagnostic tools for learning disabilities, or the uneven distribution of other comorbidities in the ADHD groups in that study, leading to more severe behavioral problems and worse executive function performance in the ADHD+LD group [21]. Therefore, considering the cognitive subtype hypothesis, despite the absence of a pure DD group in this study, it is likely that the executive function deficits in ADHD+DD are the combination or aggravation of ADHD-related and dyscalculia-related impairments [14, 31, 34]. By comparison, the deficits in scale-based executive function observed in the ADHD+DD group might primarily stem from executive function impairments associated with ADHD (a phenotype of ADHD) or shared deficits common to both ADHD and dyscalculia [14].

### 4.2 Executive Function Characteristics of ADHD Symptoms and Arithmetic Skill

In this study, we explored the relationship between executive function, symptoms, and arithmetic ability by partial correlation analysis and multiple regression analysis among the three groups. Partial correlation analysis showed that executive functions had significant correlation with ADHD symptoms and arithmetic ability. The results of multiple regression analysis demonstrated that both ADHD symptoms and arithmetic ability were affected by verbal working memory. Processing speed and scale-based WM factors significantly influenced inattention and arithmetic ability, while arithmetic ability was not influenced by the PO factor with the controlling of inattention (as shown in Tables 4,5,6 and **Supplementary Material 5**).

According to the multiple deficit model, the etiology of shared risk factors overlapping at the cognitive level leads to comorbidity symptoms [7, 14]. This is consistent with previous research that showed that processing speed and verbal working memory are crucial for both ADHD symptoms and learning abilities [27, 28, 29]. The observation of scale-based WM factors significantly influences both inattention and arithmetic ability, supporting the common deficit hypothesis. This is a novel discovery, as previous studies have rarely explored the link between scale-based executive functions and arithmetic ability in children with DD or ADHD+DD. Prior research on children with low IQ levels showed similar results [40]. Both performance-based working memory and scale-based working memory are tied to goal-directed information storage, having a similar effect on arithmetic ability [8, 12]. The findings of more severe performance-based executive impairments in the comorbidity group also support the cognitive subtype hypothesis [14, 31]. On one hand, the executive function deficits observed in ADHD+DD may result from an accumulation of impairments associated with ADHD and DD, leading to exacerbations in verbal working memory and processing speed [30, 31]. On the other hand, inhibition and cognitive flexibility uniquely predict arithmetic ability. Imaging studies also support the coexistence of two etiological hypotheses [32, 33].

This study revealed that inattention was associated with the MI, including WM, PO, and MONI, while hyperactivity/impulsivity was mainly related to the BRI, including IB, SFT, and INIT, consistent with previous research findings. Compared with performance-based executive function, these indices are more sensitive and better reflect real-life daily functioning [41, 42]. The results of this study found that although ADHD symptoms were significantly correlated with inhibition, the regression analysis showed that this relationship was no longer significant. These discrepancies may derive from the heterogeneity of ADHD, as 30%–50% of ADHD cases present executive function deficits, alongside the diverse range of executive function tasks used [18, 39]. In addition, spatial working memory in this study was not significantly associated with arithmetic ability according to the results of the final regression analysis. Spatial-visual ability is an important component of mathematical performance but it is not the most critical ability in early childhood [43]. Children with dyscalculia primarily activate the intraparietal sulcus and prefrontal cortex to complete tasks [26]. Furthermore, subtraction task involves less spatial-visual ability.

### 4.3 Underlying Cognitive Mechanism in ADHD With Developmental Dyscalculia

The role of processing speed and inhibition in differentiating individuals with ADHD+DD or ADHD-DD suggests a specific mechanism that contributes to the comorbidity. As mentioned before, processing speed is the ability to input information and convert visual stimuli into symbols [44, 45]. Visual-motor coordination and attention are required to ensure one’s working efficiency [18]. Inhibition involves the rapid suppression of interference, enabling proficient and accurate problem-solving in mathematics [18, 46]. Individuals with DD are more susceptible to task-irrelevant information [18, 46]. These two cognitive processes highlight the visual number processing and central executive function impairments in ADHD+DD. The present study did not find a differential effect of verbal working memory between ADHD and the comorbidity, which may be related to the abnormal function of the prefrontal lobe that is involved in working memory [15, 35]. Working memory is an ability to hold information in the mind and work with it mentally [8, 46]. Another reason for this is that inhibition and processing speed likely precede working memory processes as they guarantee the efficiency and accuracy of working memory [18, 46]. In fact, all executive functions work in a coordinated manner to determine goal-oriented behavior. Our results simply emphasize the key role of processing speed and inhibition for individuals with ADHD+DD.

## 5. Advantages and Limitations

The innovation and advantages of our study are that it fills a gap in the understanding of executive dysfunction in ADHD+DD among Chinese children. This study is prospective for unlocking targeted interventions for children in the future. In addition, we added scale-based executive function assessments for ADHD+DD in order to help comprehend the daily life performance of people with ADHD+DD.

This study also has certain limitations. First, the absence of children with pure dyscalculia and the presence of comorbidity with dyslexia limited further exploration of the features of executive function and the interaction of ADHD and DD. Individuals with DD have a relatively low rate of seeking medical consultation; instead, teachers are more likely to notice and identify these individuals. Second, a cross-sectional approach was unable to identify causal relationships or track developmental changes over time [47]. Therefore, future studies need to strengthen collaboration with experts in different domains. This will not only help to supplement the sample size of pure DD cases but also promote a more comprehensive assessment of these children. Such collaborations should include educational experts and psychologists who can support individualized treatment and training for children with comorbidities and ensure long-term follow-up.

## 6. Conclusion

In this study, we utilized a cross-sectional design to explore the executive function characteristics in children with ADHD+DD. Children with ADHD+DD exhibited more significant impairments in performance-based executive functions compared with children with ADHD alone. Of these, processing speed and inhibition played unique roles in the underlying mechanisms that distinguish ADHD+DD from ADHD-DD. Both the symptom dimension and arithmetic dimension have their specific predictors, and processing speed and verbal working memory were identified as candidates with shared cognitive deficits that explain the comorbidity. This finding helps us to better understand the cognitive characteristics of the comorbidity of ADHD and dyscalculia and may offer insights for future therapeutic approaches.

## Data Availability

The data and materials that support the findings of this study are available from the corresponding author upon reasonable request.

## Abbreviations

ADHD, attention-deficit/hyperactivity disorder; ADHD-C, ADHD combined type; ADHD+DD, ADHD with developmental dyscalculia; ADHD-DD, ADHD without developmental dyscalculia; ADHD-HI, ADHD predominantly hyperactive impulsive type; ADHD-I, ADHD predominantly inattention type; ADHD+LD, ADHD with learning disability; ADHD+RD, ADHD with reading disorder (dyslexia); ANOVA, one-way analysis of variance; ANCOVA, analysis of covariance; BRI, Behavioral Regulation Index; BRIEF, Behavior Rating Inventory of Executive Function; CDIS, Clinical Diagnostic Interview scale; CF, cognitive flexibility; CI, confidence interval; CI-T, color interference time; CS, complex subtraction; C-WISC-III, Chinese revised version of the Wechsler Intelligence Scale for Children, 3rd edition; DD, developmental dyscalculia; DSM-IV, Diagnostic and Statistical Manual of Mental Disorders-Fourth Edition; ECTRL, emotional control; EF, executive function; GRA, graded reading achievement; IB, inhibition; Inh, inhibition; INIT, initiation; IQ, intelligence quotient; MI, Metacognition Index; MONI, monitor; NC, normal control; OM, organization of materials; OPES, Online Psychological Experimental System; OR, odds ratio; PO, planning/organization; PS, processing speed; RD, reading disorder (dyslexia); Reydd, detail of delayed scores; Reydi, detail of immediate scores; Reysd, structure of delayed scores; Reysi, structure of immediate scores; Reyfd, forgotten detail score; Reyfs, forgotten structure score; ROCF, Rey-Osterrieth Complex Figure; R’SPM, Raven’s Standard Progressive Matrices; SD, standard deviation; SE, standard error; SFT, shifting; SI-T, semantic interference time; SS, simple subtraction; Str1t, pure Chinese character reading; Str2t, color reading; Str3t, colored Chinese character reading; Str4t, color of the colorful Chinese characters; Stroop, Stroop Color-Word Test; SWM, Spatial working memory; TMT, Trail Making Test; VWM, verbal working memory; VisualWM, visual working memory; WISC-IQ, Wechsler Intelligence Scale-intelligence quotient; WM, working memory; WMS-III, Spatial Span Test; WST, Word Semantic Test.

## Supplementary Material

Supplementary material associated with this article can be found, in the online version, at https://doi.org/10.31083/AP42712.
