# Supplementary material for: The Role of Executive Function in the Co-occurrence of ADHD and Developmental Dyscalculia in Chinese Children
Source: Alpha Psychiatry. 2025 May 28;26(3):42712. doi: 10.31083/AP42712 (PMC12231410; doi:10.31083/AP42712)
Supplement: Supplementary file 1 [file 2757-8038-26-3-42712-s1.docx]

**Supplementary Materials**

**Contents**

**Supplementary Material 1. Measurements 1**

**Supplementary Material 2. Demographic characteristics 5**

**Supplementary Material 3. Covariance analysis and post hoc analysis of performed-based executive function tasks and scale-based executive function. 5**

**Supplementary Material 4. Logistic analysis and path analysis 8 
Supplementary Material 5. Multiple regression analysis controlling for reading ability or ADHD symptoms. 9**

**Supplementary Material 1. Measurements**

1 Clinical review and diagnosis

1）Clinical diagnostic interview scale (CDIS)：Experienced pediatric psychiatrists utilized this scale to interview parents of children with ADHD for clinical diagnosis and other comorbidity evaluations. The Chinese version is well-suited to Chinese children. According to the DSM-IV, ADHD is categorized into three subtypes: (1) predominantly inattentive type (ADHD-I); (2) predominantly hyperactive-impulsive type (ADHD-HI); and (3) combined type (ADHD-C).^1^

2) Diagnosis of dyscalculia and dyslexia: All tests were computerized with the aid of the “Online Psychological Experimental System (OPES)” (http://www.dweipsy.com/lattice/).^2,3^ Confirmation of dyscalculia and/or dyslexia occurred when any of the standard scores fell below 3 out of 9.^2,4^

①Simple subtraction: This test aimed to evaluate the calculation fluency of subtraction with numbers within 20. It encompassed a total of 92 problems, with the largest minuends being 18, and differences involving single-digit numbers. Two candidate answers were present beneath each problem on the computer screen. Participants were asked to press the ‘Q’ key for the left candidate answer and the ‘P’ key for the right candidate answer. The difference between correct and incorrect answers ranged from 1 to 3. This was a time limited (2-min) task. The split-half reliability of the test was 0.96.^2,4^

②Word semantic test: This was used to assess reading fluency. The test was employed to evaluate reading fluency. Materials in the task were adapted from the language examinations used in China in recent years. The test consisted of 120 questions and had a total time limit of 5 minutes. Each question presented a sentence with a missing word, and two candidate words were beneath the sentence. Participants used the ‘P’ key or ‘Q’ key to select the answer. The reliability of the test was 0.88.^2,4^

2 Clinical assessments

1）ADHD Rating Scale-IV: This scale was developed based on the criteria of ADHD in the DSM-IV. It contains 18 questions to assess three symptoms of inattention, hyperactivity, and impulsivity across two dimensions. The Chinese version is well-suited to Chinese children. Higher scores indicate severe ADHD symptoms.^5,6^

2) Learning abilities: All tasks were web-based and are available at http://www.dweipsy.com/lattice/.^2,3^

① Complex subtraction: This reflected the participant’s arithmetic ability. The task involved numbers within 100, with a time limit of 2 minutes. Both the minuend and the subtrahend were two-digit numbers, and the differences were also two-digit numbers. The difference between correct and incorrect answers ranged from 1 to 10.^2^

② Graded reading achievement: This was used to assess reading ability. The content of test is derived from primary and middle school textbooks, as well as final exams from each semester, and covers Chinese language knowledge, comprehension, synonyms, Pinyin, etc. The format included two-choice questions and fill-in-the-blank questions. A total of 20 minutes were allowed for the test.

3 Executive function

1) Digital span test-backward: This test, designed for verbal working memory, is a component of the Chinese revised version of the Wechsler Intelligence Scale for Children, 3rd edition (C-WISC-III). This test engages the central executive by requiring the active manipulation and reordering of information. Participants were required to repeat a sequence of digits in reverse order. Two sequences of each length were presented. The list length was increased by one number if participants correctly repeated either of the sequences. They had only one chance to repeat the given sequence. The score of the test was recorded as the highest number of sequences correctly repeated.^7,8^

2) Coding test: This task was designed to assess the function of processing speed and is also a component of the C-WISC-III. Participants were required to convert shapes into specified symbols as quickly as possible within 150 seconds, after a 10-sample practice session.^7,9^

3) Spatial Span Test (WMS-III): This test was used to assess spatial working memory capacity. The participants were presented with 10 cubes fixed to a checkboard-sized board and requested to reproduce a sequence of spatial locations in the correct order. After the examiners tapped the cubes, participants had to recall the sequence in both forward and backward order by tapping the cubes. The rules were the same as for the digital span test.^10^

4) Stroop Color-Word Test (Stroop): This test assessed inhibition ability through four tasks including character reading (str1t), color reading (str2t), colored Chinese characters reading (str3t), and color of the colorful Chinese characters (str4t) quickly and accurately within a limited time. The color interference time (CI-T) was equal to the times of the str3t minus the times of the str1t. The semantic interference time (SI-T) was equal to the times of the str4t minus the times of the str2t. The longer the time taken to complete the test, the poorer the inhibitory function.^11,12^

5) Trail Making Test (TMT): This test comprises two tasks designed to assess cognitive flexibility, working memory, and processing speed. In the TMT-A, participants were asked to connect a series of numbers in ascending order as quickly as possible. In the TMT-B, participants were required to connect numbers and letters alternatively in ascending order. The time taken to complete each part was recorded. Shifting time is equal to TMT-B times minus TMT-A times. Longer time indicates worse performance in this test.^13,14^

6) Rey-Osterrieth Complex Figure (ROCF): This test evaluated visual working memory. Participants were given 30 seconds to memorize a specific figure and then were asked to reproduce the figure on a blank piece of paper as accurately as possible. They were requested to recall the figure after 30 minutes. The structure of immediate scores (Reysi), the detail of immediate scores (Reydi), the structure of delayed scores (Reysd), and the detail of delayed scores (Reydd) were recorded. The forgotten structure score (Reyfs) was calculated by subtracting the structure of the delayed score from the structure of the immediate score. Similarly, the forgotten detail score (Reyfd) was obtained by subtracting immediate detail from delayed detail. Higher scores indicate better performance on visual working memory.^12,15^

7）Behavior Rating Inventory of Executive Function (BRIEF): This executive function assessment was based on parent-rating questionnaire developed by Gioia. The questionnaire was used to evaluate ecological executive functions for children aged 6–18 years based on their daily life behavior. The questionnaire contains eight domains: inhibition (IB), shifting (SFT) , emotional control (ECTRL)，initiating (INIT), working memory (WM), planning/organization (PO), organization of material (OM), and monitor (MONI). According to the frequency of occurrence, scores ranged from 1=never to 3=often. Higher scores indicate more severe executive function impairment.^16,17^

4 Intelligence test

1) China-Wechsler Intelligence Scale 3rd edition (C-WISC-III): This assessed the general intellectual level of children aged 6–16 years, excluding those with intellectual disabilities. The Chinese version is well-suited to Chinese children. Well-trained examiners administered the test following standard instructions. The split-half correlations were around 0.8 and test-retest reliabilities ranged from 0.60 to 0.8.^7,18^

2) Raven’s Standard Progressive Matrices (R’SPM): This non-verbal intelligence test assessed reasoning ability. The R’SPM is not constrained by language or cultural knowledge, making it suitable for individuals with diverse backgrounds and language proficiencies. The test consists of 60 questions, where participants identify patterns in given graphs and select an appropriate pattern to complete the missing part of the graph. The reliability of the SPM was 0.88.^19^

**References for Measurements**

1. Yang L, Wang YF, Qian QJ, Biederman J, Faraone SV. DSM-IV subtypes of ADHD in a Chinese outpatient sample. *J Am Acad Child Adolesc Psychiatry*. 2004;43(3):248-250.

2. Wei W, Lu H, Zhao H, Chen C, Dong Q, Zhou X. Gender differences in children's arithmetic performance are accounted for by gender differences in language abilities. *Psychol Sci*. 2012;23(3):320-330.

3. Zhou X, Hu Y, Yuan L, Gu T, Li D. Visual form perception predicts 3-year longitudinal development of mathematical achievement. *Cogn Process*. 2020;21(4):521-532.

4. Cheng D, Xiao Q, Chen Q, Cui J, Zhou X. Dyslexia and dyscalculia are characterized by common visual perception deficits. *Dev Neuropsychol*. 2018;43(6):497-507.

5. Su YE, Wang H, Geng YG, et al. Parent Ratings of ADHD Symptoms in Chinese Urban Schoolchildren: Assessment With the Chinese ADHD Rating Scale-IV: Home Version. *J Atten Disord*. 2015;19(12):1022-1033.

6. Hebert GW. The psychometric characteristics of the ADHD Rating Scale-IV. In: ProQuest Dissertations Publishing; 1998.

7. Wang X. Factor Analysis of the WISC-R and WISC-CR: A Comparison across Ages between American and Chinese Samples. In: 1992.

8. Giofre D, Stoppa E, Ferioli P, Pezzuti L, Cornoldi C. Forward and backward digit span difficulties in children with specific learning disorder. *J Clin Exp Neuropsychol*. 2016;38(4):478-486.

9. Shanahan MA, Pennington BF, Yerys BE, et al. Processing speed deficits in attention deficit/hyperactivity disorder and reading disability. *J Abnorm Child Psychol*. 2006;34(5):585-602.

10. Brown LA. Spatial-Sequential Working Memory in Younger and Older Adults: Age Predicts Backward Recall Performance within Both Age Groups. *Front Psychol*. 2016; 7:1514.

11. Scarpina F, Tagini S. The Stroop Color and Word Test. *Front Psychol*. 2017; 8:557.

12. Shuai L, Wang YF. [Executive function characteristic in boys with attention deficit hyperactivity disorder comorbid learning disabilities]. *Beijing Da Xue Xue Bao Yi Xue Ban*. 2007;39(5):526-530.

13. Delis DC, Kaplan E, Kramer JH. Delis-Kaplan executive function system (D-KEFS). 2001.

14. Arbuthnott K, Frank J. Trail making test, part B as a measure of executive control: validation using a set-switching paradigm. *J Clin Exp Neuropsychol*. 2000;22(4):518-528.

15. Seidman LJ, Benedict KB, Biederman J, Bernstein JH, Faraone SV. Performance of Children with ADHD on the Rey-Osterrieth Complex Figure: A Pilot Neuropsychological Study. *J Child Psychol Psychiatry*. 2010;36(8):1459-1473.

16. Ying Q, Beijing, China. [Reliability and validity of behavior rating scale of executive function parent form for school age children in China]. *Chinese Mental Health Journal*. 2009;39(3):277.

17. Gioia GA, Isquith PK, Retzlaff PD, Espy KA. Confirmatory factor analysis of the Behavior Rating Inventory of Executive Function (BRIEF) in a clinical sample. *Child Neuropsychol*. 2002;8(4):249-257.

18. Gong Y, Cai T. Manual of Wechsler Intelligence Scale for Children, Chinese revision (C-WISC). 1993.

19. Zhang HC, Wang XP. Standardization research on Raven's Standard Progressive Matrices in China. Xin Li Xue Bao. 1989; 21:3-11.

**Supplementary Material 2. Demographic characteristics**

| Table 1 Other comorbidities characteristic of the ADHD+DD group and ADHD-DD group | | | |
| --- | --- | --- | --- |
| Object | ADHD+DD | ADHD-DD | *P-value* |
|  | （n=150） | （n=357） |  |
| Total (%) | 42 (28.00) | 111 (31.09) | 0.489 |
| ODD (%) | 18 (12.00) | 56 (15.69) | 0.283 |
| CD (%) | 0 (0.00) | 3 (0.084) | 0.559 |
| DBD-NOS (%) | 2 (1.30) | 8 (2.24) | 0.748 |
| SP1(%) | 8 (5.30) | 16 (4.48) | 0.68 |
| SP2(%) | 0 (0.00) | 2 (0.06) | 1.000 |
| GAD (%) | 0 (0.00) | 4 (1.12) | 0.452 |
| Dysthymia (%) | 1 (0.67) | 10 (2.80) | 0.241 |
| MDD (%) | 0 (0.00) | 11 (3.08) | 0.066 |
| Tics (%) | 21 (14.00) | 37 (10.36) | 0.24 |
| OCD (%) | 2 (1.30) | 0 (0.00) | 0.087 |

Abbreviations: ADHD=attention-deficit/hyperactivity disorder; DD=developmental dyscalculia; ODD=oppositional defiant disorder; CD=conduct disorder; DBD-NOS=disruptive behavior disorder-not otherwise specified; SP1=specific phobia; SP2=social phobia; GAD=generalized anxiety disorder; MDD=major depressive disorder; OCD=obsessive-compulsive disorder.

**Supplementary Material 3. Covariance analysis and post hoc analysis of performed-based executive function tasks and scale-based executive function**

| Table 2 Between-group comparisons of performance-based executive function tasks | | | | | | | | | |
| --- | --- | --- | --- | --- | --- | --- | --- | --- | --- |
| Tasks | | 1.ADHD+DD  （n=150） | 2.ADHD-DD  （n=357） | 3.NC  （n=130） | *P*-value | *post hoc* | 1 vs 2 | 1 vs 3 | 2 vs 3 |
| WISC-IQ | Backward | 2.20±0.03 | 2.30±0.02 | 2.45±0.03 | <0.001 | 3>2>1 | 0.002 | <0.001 | <0.001 |
|  | Coding test | 7.89±0.27 | 9.84±0.17 | 11.24±0.29 | ＜0.001 | 3>2>1 | <0.001 | <0.001 | <0.001 |
| WMS-III | Forward | 2.64±0.03 | 2.73±0.02 | 2.99±0.03 | <0.001 | 3>2>1 | 0.018 | <0.001 | <0.001 |
|  | Backward | 5.02±0.16 | 5.39±0.10 | 7.03±0.18 | <0.001 | 3>2,1 | 0.166 | <0.001 | <0.001 |
|  | Total | 11.15±0.25 | 11.97±0.16 | 15.09±0.28 | <0.001 | 3>2>1 | 0.018 | <0.001 | <0.001 |
| ROCF | Reysi | 2.28±0.17 | 2.80±0.11 | 3.46±0.19 | <0.001 | 3>2>1 | 0.031 | <0.001 | 0.007 |
|  | Reydi | 2.83±0.08 | 3.03±0.05 | 3.42±0.09 | <0.001 | 3>2,1 | 0.110 | <0.001 | <0.001 |
|  | Reysd | 2.31±0.16 | 2.92±0.10 | 3.46±0.18 | <0.001 | 3>2>1 | 0.005 | <0.001 | 0.025 |
|  | Reydd | 2.73±0.08 | 2.98±0.05 | 3.37±0.09 | <0.001 | 3>2>1 | 0.022 | <0.001 | <0.001 |
|  | Reyfs | 0.06±0.08 | -0.03±0.05 | 0.00±0.08 | 0.627 | - |  |  |  |
|  | Reyfd | 0.55±0.24 | 0.23±0.15 | 0.32±0.26 | 0.359 | - |  |  |  |
| TMT | TMT-A | 1.78±0.01 | 1.74±0.01 | 1.65±0.01 | <0.001 | 3<2<1 | 0.006 | <0.001 | <0.001 |
|  | TMT-B | 2.29±0.01 | 2.19±0.01 | 2.12±0.02 | <0.001 | 3<2<1 | <0.001 | <0.001 | 0.001 |
|  | Shifting time | 11.97±0.24 | 10.38±0.15 | 9.80±0.26 | <0.001 | 3,2<1 | <0.001 | <0.001 | 0.164 |
| Stroop | str1t | 1.33±0.01 | 1.25±0.01 | 1.21±0.01 | <0.001 | 3<2<1 | <0.001 | <0.001 | 0.001 |
|  | str2t | 1.46±0.01 | 1.38±0.01 | 1.34±0.01 | <0.001 | 3<2<1 | <0.001 | <0.001 | <0.001 |
|  | str3t | 1.44±0.01 | 1.36±0.01 | 1.30±0.01 | <0.001 | 3<2<1 | <0.001 | <0.001 | <0.001 |
|  | str4t | 7.77±0.09 | 7.17±0.05 | 6.47±0.10 | <0.001 | 3<2<1 | <0.001 | <0.001 | <0.001 |
|  | CI-T | 4.70±0.06 | 4.62±0.04 | 4.47±0.07 | 0.061 | - |  |  |  |
|  | SI-T | 6.91±0.08 | 6.61±0.05 | 6.07±0.10 | <0.001 | 3<2<1 | 0.007 | <0.001 | <0.001 |

Note: ^a^Estimated marginal means (EMM) adjusted for covariates, presented in the table.

Abbreviations: ADHD+DD=ADHD with developmental dyscalculia; ADHD-DD=ADHD without developmental dyscalculia; NC=normal control; WISC-IQ=Wechsler Intelligence Scale-intelligence quotient; WMS-III=Spatial Span Test; ROCF=Rey-Osterrieth Complex Figure; Reysi=structure of immediate scores; Reydi=detail of immediate scores; Reysd=structure of delayed scores; Reydd=detail of delayed scores; Reyfs=forgotten structure score; Reyfd=forgotten detail score; TMT=Trail Making Test; Stroop=Stroop Color-Word Test; Str1t=Pure Chinese character reading; Str2t=Color reading; Str3t=Colored Chinese character reading; Str4t=Color of the colorful Chinese characters; CI-T=color interference time; SI-T=semantic interference time.

| Table 3 Between-group comparisons of performance-based executive function factors | | | | | | | | |
| --- | --- | --- | --- | --- | --- | --- | --- | --- |
| Projects | 1. ADHD+DD   （n=150） | 1. ADHD-DD   （n=357） | 1. NC   （n=130） | *P*-value | *post hoc* | 1 vs 2 | 1 vs 3 | 2 vs 3 |
| SWM | -0.31^a^±0.06 | -0.11±0.04 | 0.66±0.07 | <0.001 | 3>2>1 | 0.016 | <0.001 | <0.001 |
| VisualWM | -0.24±0.07 | -0.01±0.04 | 0.31±0.08 | <0.001 | 3>2>1 | 0.012 | <0.001 | 0.001 |
| CF | 0.36±0.06 | 0.00±0.04 | -0.41±0.06 | <0.001 | 3<2<1 | <0.001 | <0.001 | <0.001 |
| Inh | 0.52±0.06 | -0.05±0.04 | -0.50±0.07 | <0.001 | 3<2<1 | <0.001 | <0.001 | <0.001 |

Note: ^a^Estimated marginal means (EMM) adjusted for covariates, presented in the table.

Abbreviations: ADHD+DD=ADHD with developmental dyscalculia; ADHD-DD=ADHD without developmental dyscalculia; NC=normal control; SWM=spatial working memory; VisualWM=visual working memory; CF=cognitive flexibility; Inh=inhibition.

| Table 4 Between-group comparisons of scale-based executive function | | | | | | | | |
| --- | --- | --- | --- | --- | --- | --- | --- | --- |
| Factors | 1. ADHD+DD   （n=150） | 1. ADHD-DD   （n=357） | 1. NC   （n=130） | *P*-value | *post hoc* | 1 vs 2 | 1 vs 3 | 2 vs 3 |
| IB | 1.25^a^±0.01 | 1.25±0.01 | 1.11±0.01 | <0.001 | 1,2>3 | 1.000 | <0.001 | <0.001 |
| SFT | 2.52±0.02 | 2.52±0.01 | 2.38±0.02 | <0.001 | 1,2>3 | 1.000 | <0.001 | <0.001 |
| ECTRL | 1.19±0.01 | 1.20±0.01 | 1.12±0.01 | <0.001 | 1,2>3 | 1.000 | <0.001 | <0.001 |
| INIT | 15.26±0.26 | 15.01±0.16 | 11.79±0.28 | <0.001 | 1,2>3 | 1.000 | <0.001 | <0.001 |
| WM | 22.41±0.28 | 21.90±0.18 | 15.76±0.31 | <0.001 | 1,2>3 | 0.327 | <0.001 | <0.001 |
| PO | 0.39±0.07 | 0.22±0.04 | -1.04±0.08 | <0.001 | 1,2>3 | 0.113 | <0.001 | <0.001 |
| OM | 0.96±0.08 | 0.20±0.05 | -0.70±0.08 | <0.001 | 1,2>3 | 0.799 | <0.001 | <0.001 |
| MONI | 0.26±0.07 | 0.23±0.04 | -0.97±0.08 | <0.001 | 1,2>3 | 1.000 | <0.001 | <0.001 |

Note: ^a^Estimated marginal means (EMM) adjusted for covariates, presented in the table.

Abbreviations: ADHD+DD=ADHD with developmental dyscalculia; ADHD-DD=ADHD without developmental dyscalculia; NC=normal control; IB=inhibition; SFT=shifting; ECTRL=emotional control; INIT=initiating; WM=working memory; PO=planning/organization; OM=organization of material; MONI=monitor.

**Supplementary Material 4. Logistic analysis and path analysis**

| Table 5 Significant effects on ADHD groups identified by logistic regression analysis | | | | | | |
| --- | --- | --- | --- | --- | --- | --- |
| Model | Subject | B | SE | *P-value* | OR | 95% CI |
|  |  |  |  |  |  |  |
| Performance-based EF | Constant | -0.07 | 1.34 | 0.956 | 0.93 |  |
|  | Age | 0.02 | 0.01 | 0.003 | 1.02 | 1.01; 1.03 |
|  | Raven | -0.04 | 0.02 | 0.012 | 0.96 | 0.94; 0.99 |
|  | VWM | -0.54 | 0.40 | 0.179 | 0.59 | 0.27; 1.28 |
|  | SWM | -0.10 | 0.16 | 0.511 | 0.90 | 0.66; 1.23 |
|  | CF | 0.32 | 0.19 | 0.084 | 1.38 | 0.96; 1.98 |
|  | Inh | 0.69 | 0.18 | <0.001 | 2.00 | 1.42; 2.81 |
|  | VisualWM | -0.20 | 0.14 | 0.158 | 0.82 | 0.62; 1.08 |
|  | PS | -0.11 | 0.04 | 0.004 | 0.90 | 0.84; 0.97 |

Note: n=507(ADHD-DD=357, ADHD+DD=150).

Abbreviation: SE=standard error; OR=odds ratio; Raven=raw score of Raven’s Standard Progressive Matrices; EF=executive function; VWM=verbal working memory; SWM=spatial working memory; VisualWM=visual working memory; PS=processing speed; Inh=inhibition; CF=cognitive flexibility.

| Table 6 Standardized total effect, direct effect, and indirect effect identified by path analysis | | |
| --- | --- | --- |
|  | PS | Inh |
| Total effect | -0.15 | 0.34 |
| 95% CI | -0.24; -0.05 | 0.22; 0.45 |
| Direct effect | -0.15 | 0.34 |
| 95% CI | -0.24; -0.05 | 0.23; 0.44 |
| Indirect effect | <0.001 | <0.001 |
| 95% CI | / | / |

Note: n=507 (ADHD-DD=357, ADHD+DD=150).

Abbreviations: PS=processing speed; Inh=inhibition; CI=confidence interval.

**Supplementary Material 5. Multiple regression analysis controlling for reading ability or ADHD symptoms.**

| Table 7 Multiple regression analysis of the effect on arithmetic ability with controlling for reading ability | | | | | | | | |
| --- | --- | --- | --- | --- | --- | --- | --- | --- |
| Subject | Step 1 | | Performance-based  EF | Step 2-1 | | Scale-based  EF | Step 2-2 | |
|  | *β* | *P-value* |  | *β* | *P-value* |  | *β* | *P-value* |
| Constant |  |  | Constant |  | 0.031 | Constant |  | 0.129 |
| Gender | 0.09 | 0.005 | Gender | 0.05 | 0.127 | Gender | 0.07 | 0.036 |
| Age | 0.18 | <0.001 | Age | 0.07 | 0.107 | Age | 0.21 | <0.001 |
| Raven | 0.18 | <0.001 | Raven | 0.07 | 0.083 | Raven | 0.17 | <0.001 |
| GRA | 0.37 | <0.001 | GRA | 0.26 | <0.001 | Reading | 0.34 | <0.001 |
|  |  |  | VWM | 0.10 | 0.012 | IB | 0.03 | 0.543 |
|  |  |  | SWM | 0.07 | 0.103 | SFT | 0.04 | 0.377 |
|  |  |  | Visual WM | 0.02 | 0.656 | ECTRL | -0.02 | 0.658 |
|  |  |  | PS | 0.11 | 0.003 | INIT | 0.01 | 0.910 |
|  |  |  | Inh | -0.10 | 0.041 | WM | -0.14 | 0.009 |
|  |  |  | CF | -0.11 | 0.020 | PO | -0.11 | 0.041 |
|  |  |  |  |  |  | OM | 0.04 | 0.332 |
|  |  |  |  |  |  | MONI | 0.09 | 0.107 |
|  |  |  |  |  |  |  |  |  |
| R² | 0.415 |  |  | 0.472 |  |  | 0.436 |  |
| Corrected R² | 0.411 |  |  | 0.464 |  |  | 0.425 |  |
| F | <0.001 |  |  | <0.001 |  |  | <0.001 |  |
| △R² | 0.415 |  |  | 0.057 |  |  | 0.021 |  |
| △F | <0.001 |  |  | <0.001 |  |  | 0.004 |  |

Note: n=637（ADHD-DD=357, ADHD+DD=150, and NC=130）.

Abbreviations: β=standardized coefficients; EF=executive function; Raven=raw score of Raven’s Standard Progressive Matrices; GRA=graded reading achievement; VWM=verbal working memory; SWM=spatial working memory; VisualWM=visual working memory; PS=processing speed; Inh=inhibition; CF=cognitive flexibility; IB=inhibition; SFT=shifting; ECTRL=emotional control; INIT=initiating; WM=working memory; PO=planning/organization; OM=organization of material; MONI=monitor.

| Table 8 Multiple regression analysis of the effect on arithmetic ability controlling for inattention symptoms | | | | | | | | |
| --- | --- | --- | --- | --- | --- | --- | --- | --- |
| Subject | Step 1 | | Performance-based  EF | Step 2-1 | | Scale-based  EF | Step 2-2 | |
|  | *β* | *P-value* |  | *β* | *P-value* |  | *β* | *P-value* |
| Constant |  | <0.001 | Constant |  | 0.030 | Constant |  | 0.115 |
| Gender | 0.07 | <0.001 | Gender | 0.03 | 0.287 | Gender | 0.07 | 0.051 |
| Age | 0.35 | 0.046 | Age | 0.16 | <0.001 | Age | 0.37 | <0.001 |
| Raven | 0.28 | <0.001 | Raven | 0.13 | 0.002 | Raven | 0.27 | <0.001 |
| Inattention | -0.19 | <0.001 | Inattention | -0.10 | 0.005 | Inattention | -0.18 | <0.001 |
|  |  |  | VWM | 0.11 | 0.005 | IB | 0.01 | 0.806 |
|  |  |  | SWM | 0.05 | 0.211 | SFT | -0.12 | 0.473 |
|  |  |  | Visual WM | 0.04 | 0.338 | ECTRL | 0.03 | 0.811 |
|  |  |  | PS | 0.11 | 0.003 | INIT | 0.01 | 0.880 |
|  |  |  | Inh | -0.13 | 0.006 | WM | -0.12 | 0.038 |
|  |  |  | CF | -0.12 | 0.014 | PO | -0.10 | 0.078 |
|  |  |  |  |  |  | OM | 0.07 | 0.090 |
|  |  |  |  |  |  | MONI | 0.13 | 0.030 |
|  |  |  |  |  |  |  |  |  |
| R² | 0.378 |  |  | 0.450 |  |  | 0.394 |  |
| Corrected R² | 0.374 |  |  | 0.441 |  |  | 0.383 |  |
| F | <0.001 |  |  | <0.001 |  |  | <0.001 |  |
| △R² | 0.378 |  |  | 0.073 |  |  | 0.017 |  |
| △F | <0.001 |  |  | <0.001 |  |  | 0.030 |  |

Note: n=637（ADHD-DD=357, ADHD+DD=150, and NC=130）.

Abbreviations: β=standardized coefficients; EF=executive function; Raven=raw score of Raven’s Standard Progressive Matrices; VWM=verbal working memory; SWM=spatial working memory; VisualWM=visual working memory; PS=processing speed; Inh=inhibition; CF=cognitive flexibility; IB=inhibition; SFT=shifting; ECTRL=emotional control; INIT=initiating; WM=working memory; PO=planning/organization; OM=organization of material; MONI=monitor.

| Table 9 Multiple regression analysis of the effect on arithmetic ability controlling for hyperactivity/impulsivity symptoms | | | | | | | | |
| --- | --- | --- | --- | --- | --- | --- | --- | --- |
| Subject | Step 1 | | Performance-based  EF | Step 2-1 | | Scale-based  EF | Step 2-2 | |
|  | *β* | *P-value* |  | *β* | *P-value* |  | *β* | *P-value* |
| Constant |  | <0.001 | Constant |  | 0.024 | Constant |  | 0.010 |
| Gender | 0.09 | 0.008 | Gender | 0.04 | 0.271 | Gender | 0.07 | 0.049 |
| Age | 0.32 | <0.001 | Age | 0.13 | 0.004 | Age | 0.35 | <0.001 |
| Raven | 0.31 | <0.001 | Raven | 0.12 | 0.002 | Raven | 0.28 | <0.001 |
| HI | -0.10 | 0.002 | HI | -0.08 | 0.017 | HI | -0.14 | <0.001 |
|  |  |  | VWM | 0.11 | 0.004 | IB | 0.10 | 0.104 |
|  |  |  | SWM | 0.07 | 0.103 | SFT | 0.03 | 0.494 |
|  |  |  | Visual WM | 0.04 | 0.364 | ECTRL | -0.01 | 0.768 |
|  |  |  | PS | 0.12 | 0.001 | INIT | -0.01 | 0.856 |
|  |  |  | Inh | -0.14 | 0.004 | WM | -0.18 | <0.001 |
|  |  |  | CF | -0.13 | 0.009 | PO | -0.13 | 0.018 |
|  |  |  |  |  |  | OM | 0.07 | 0.101 |
|  |  |  |  |  |  | MONI | 0.10 | 0.091 |
|  |  |  |  |  |  |  |  |  |
| R² | 0.355 |  |  | 0.448 |  |  | 0.390 |  |
| Corrected R² | 0.351 |  |  | 0.439 |  |  | 0.378 |  |
| F | <0.001 |  |  | <0.001 |  |  | <0.001 |  |
| △R² | 0.355 |  |  | 0.093 |  |  | 0.035 |  |
| △F | <0.001 |  |  | <0.001 |  |  | <0.001 |  |

Note: n=637（ADHD-DD=357, ADHD+DD=150, and NC=130）.

Abbreviations: β=standardized coefficients; EF=executive function; Raven=raw score of Raven’s Standard Progressive Matrices; HI=hyperactivity-impulsivity; VWM=verbal working memory; SWM=spatial working memory; VisualWM=visual working memory; PS=processing speed; Inh=inhibition; CF=cognitive flexibility; IB=inhibition; SFT=shifting; ECTRL=emotional control; INIT=initiating; WM=working memory; PO=planning/organization; OM=organization of material;MONI=monitor.
